# Two Distinct Repressive Mechanisms for Histone 3 Lysine 4 Methylation through Promoting 3′-End Antisense Transcription

**DOI:** 10.1371/journal.pgen.1002952

**Published:** 2012-09-20

**Authors:** Thanasis Margaritis, Vincent Oreal, Nathalie Brabers, Laetitia Maestroni, Adeline Vitaliano-Prunier, Joris J. Benschop, Sander van Hooff, Dik van Leenen, Catherine Dargemont, Vincent Géli, Frank C. P. Holstege

**Affiliations:** 1 Molecular Cancer Research, University Medical Center Utrecht, Utrecht, The Netherlands; 2 Marseille Cancer Research Center (CRCM), U1068 Inserm, UMR7258 CNRS, Aix-Marseille Université, Institut Paoli-Calmettes, Marseille, France; 3 Institut Jacques Monod, Université Paris Diderot, CNRS, Paris, France; University of California San Francisco, United States of America

## Abstract

Histone H3 di- and trimethylation on lysine 4 are major chromatin marks that correlate with active transcription. The influence of these modifications on transcription itself is, however, poorly understood. We have investigated the roles of H3K4 methylation in *Saccharomyces cerevisiae* by determining genome-wide expression-profiles of mutants in the Set1 complex, COMPASS, that lays down these marks. Loss of H3K4 trimethylation has virtually no effect on steady-state or dynamically-changing mRNA levels. Combined loss of H3K4 tri- and dimethylation results in steady-state mRNA upregulation and delays in the repression kinetics of specific groups of genes. COMPASS-repressed genes have distinct H3K4 methylation patterns, with enrichment of H3K4me3 at the 3′-end, indicating that repression is coupled to 3′-end antisense transcription. Further analyses reveal that repression is mediated by H3K4me3-dependent 3′-end antisense transcription in two ways. For a small group of genes including *PHO84*, repression is mediated by a previously reported trans-effect that requires the antisense transcript itself. For the majority of COMPASS-repressed genes, however, it is the process of 3′-end antisense transcription itself that is the important factor for repression. Strand-specific qPCR analyses of various mutants indicate that this more prevalent mechanism of COMPASS-mediated repression requires H3K4me3-dependent 3′-end antisense transcription to lay down H3K4me2, which seems to serve as the actual repressive mark. Removal of the 3′-end antisense promoter also results in derepression of sense transcription and renders sense transcription insensitive to the additional loss of *SET1*. The derepression observed in COMPASS mutants is mimicked by reduction of global histone H3 and H4 levels, suggesting that the H3K4me2 repressive effect is linked to establishment of a repressive chromatin structure. These results indicate that in *S. cerevisiae*, the non-redundant role of H3K4 methylation by Set1 is repression, achieved through promotion of 3′-end antisense transcription to achieve specific rather than global effects through two distinct mechanisms.

## Author Summary

In eukaryotes, DNA is packaged together with histones into nucleosomes. This packaging has a repressive role on gene expression. The N-termini of histones are subject to multiple modifications that affect DNA–dependent processes. The histone modification that has been predominantly linked with active transcription in all eukaryotes is histone H3 lysine 4 (H3K4) methylation. Here we investigate the functional effects of each H3K4 methylation state on transcription. Removal of the mark that is most characteristic for transcription, H3K4 trimethylation, has no effect on coding gene expression, in steady-state or dynamically changing conditions. Combined loss of H3K4 tri- and di-methylation does have an effect and leads to loss of repression of specific genes, the opposite of what is expected for global marks of active genes. The affected genes have antisense transcription. We show that there are two separate mechanisms through which H3K4 methylation represses transcription of protein-coding genes, one through antisense transcripts and one through the process of antisense transcription. In summary, we show how a general mark of active transcription can have specific repressive effects that are themselves also linked to repression through nucleosomes.

## Introduction

Packaging of eukaryotic DNA with histones has a generally repressive effect on transcription [1]. Histones themselves are subject to a variety of post-translational modifications, such as acetylation, methylation and ubiquitinylation. These modifications correlate with specific states of transcription, as well as with the activity of other DNA-linked processes, such as chromosome segregation and DNA repair [2], [3]. Among the epigenetic marks, histone methylation has been extensively associated with both activation and repression of genes in euchromatic and heterochromatic regions respectively [4]. Methylation of histone H3 on lysine 4 (H3K4) for example, has been linked to transcriptional activation in many eukaryotic species. Vertebrates possess several H3K4 methyltransferases related to the SET domain of yeast Set1 and Drosophila Trx (MLL family) [5]. These methyltransferases are responsible for mono- (H3K4me1), di- (H3K4me2) and trimethylation (H3K4me3) of H3K4 [6]. Di- and trimethylation of H3K4 is generally restricted to euchromatin and genome-wide studies in metazoan cells have revealed high levels of histone acetylation and H3K4 methylation in promoter regions of active genes [7], [8], [9], [10], [11]. H3K4me2 and H3K4me3 are thought to facilitate transcription through the recruitment of general transcription factors [12] and cofactors [13] or by preventing repressors from binding to chromatin [14]. The precise mechanism through which the various H3K4 methylation states contribute to control of gene expression are not fully understood.

In *Saccharomyces cerevisiae*, H3K4 methylation is carried out by the Set1 complex, COMPASS [15], which is composed of the catalytic subunit Set1 and at least six other components (Swd1, Swd2, Swd3, Bre2, Sdc1 and Spp1) [16], [17], [18], [19]. Loss or inactivation of individual subunits differentially affects the methylation state of H3K4. Swd1, Swd2 and Swd3 are required for COMPASS stability and their disruption affects all three H3K4 methylation states. Bre2 and Sdc1 promote the efficient di- and trimethylation of H3K4, while inactivation of Spp1 only affects H3K4 trimethylation [20], [21], [22]. In addition, monoubiquitylation of Swd2 has recently been shown to mediate the trans-tail process between H2B ubiquitylation and H3K4 trimethylation, by controlling the recruitment of the Spp1 subunit [23]. Set1 has been found to be predominantly associated with the coding regions of highly transcribed RNA polymerase II genes and the presence of trimethylated H3K4 correlates with Set1 occupancy [24] and transcription rate [25]. Genome-wide studies in yeast indicate that active transcription is characteristically accompanied by histone H3K4 trimethylation at the 5′-end of genes and by H3K4 dimethylation and monomethylation at nucleosomes positioned further downstream in the transcription unit [25].

Although H3K4 trimethylation has been linked to transcription initiation and elongation in yeast [6], [21], [26], its precise role in transcription as well as the role of H3K4 mono- and dimethylation remain poorly understood. This is in part because previous genome-wide analyses of the effects of H3K4 methylation loss have yielded conflicting results [6], [27], [28], [29] [30]. While two studies suggested a global reduction in transcription when H3K4 methylation is abolished [27], [28], a third study reported and focused on only 480 very marginally down-regulated genes, even though twice as many genes were observably upregulated upon applying the same selection criteria [6]. The most recent study also reported roughly 300 genes up-regulated and 100 down-regulated [30]. A more statistically stringent study that included adequate replicate experiments showed that 200 genes become up-regulated upon loss of *SET1*, with virtually no down-regulation observed [29], suggesting that H3K4 methylation may actually play a more prominent role in repression than in activation of protein-coding genes.

Recently, a form of RNA-mediated transcriptional repression has been reported in *S. cerevisiae*, that is independent of the RNAi machinery which is absent from budding yeast. Ty1, *PHO84* and *GAL1/10* expression have been shown to be regulated by antisense RNA transcription [31], [32], [33]. For *PHO84*, it was found that expression of *PHO84* antisense RNA from an ectopic *PHO84* gene copy was able to trigger silencing of the endogenous *PHO84* gene [34]. Production of the *PHO84* antisense RNA was found to be positively regulated by Set1 [34] potentially linking H3K4 methylation to non-coding RNA (ncRNA) regulation. Genome-wide analysis has recently revealed the existence of hundreds of previously uncharacterized ncRNAs in mammals [35], [36], [37], [38] and in yeast [39], [40], that either stably exist or are rapidly degraded by the RNA surveillance pathway. Strikingly, most of these newly identified transcripts initiate from nucleosome-free regions associated with bidirectional promoters of protein-coding genes or regions in the body or close to the 3′-ends of protein-coding genes [40]. Regulation of ncRNAs is far from understood.

Here we present an extensive genome-wide analysis that discriminates between the roles of the different H3K4 methylation states. While preventing H3K4 trimethylation on its own has no effect on mRNA expression of coding genes, 1% of coding genes are derepressed upon combined loss of di- and trimethylation. Further analyses indicate distinct roles for these two marks in repression of coding genes through mechanisms that are mediated through 3′-end antisense transcription.

## Results

### Loss of H3K4 dimethylation correlates with increased expression of a subset of genes

**Figure 1:**
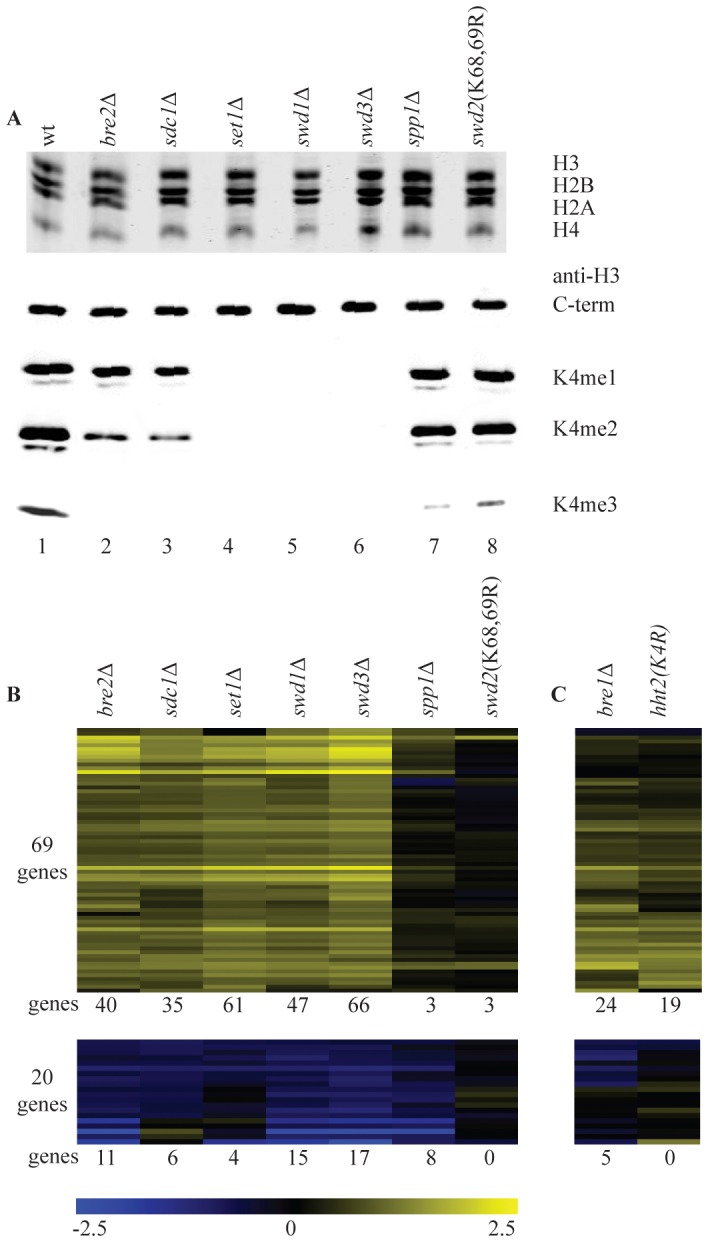
Loss of H3K4 di- and trimethylation results in upregulation of a subset of genes. (A) Commassie-stained gel of purified histones from the indicated strains (top) and western blots with antibodies directed against H3 carboxy-terminus and the different H3K4 methylation states (bottom). (B) Hierarchical clustering of all genes with significantly changed mRNA expression (p-value less than 0.01 and fold-change versus wild-type more than 1.7) in at least two COMPASS mutants. Fold-change of mRNA expression in mutant versus wild-type is indicated by the colour bar as log_2_ values. Number of genes below each heatmap correspond to the genes called significant in each mutant. (C) Genes depicted in the same order as in B for the H3K4R point mutant and *bre1*Δ.

Previous genome-wide analyses of the effects of losing H3K4 methylation [6], [27], [28], [29] [30] focused on loss of all three H3K4 methylation states simultaneously, either through deletion of the gene that codes for the H3K4 methyltransferase, *SET1* or through substitution of H3K4 with alanine or arginine. To investigate whether there are separate roles for H3K4 mono-, di- and trimethylation, we made use of the fact that mutating different components of the Set1 complex, COMPASS, results in different methylation states. First, the methylation status of H3K4 was assessed in strains with deletions of the non-essential members of the complex, in the single genetic background used for this study (BY4741). An additional strain was included that carries a mutation that prevents monoubiquitylation of the essential subunit Swd2 (swd2K68,69R), resulting in a severe reduction of H3K4me3 [23]. Histones were purified from each strain and their H3K4 methylation status was checked with antibodies specific for each methylated state (Figure 1A). As expected from previous results (see the introduction), deletion of *SET1*, *SWD1* or *SWD3* abolishes mono-, di- and trimethylation of H3K4. Deletion of *BRE2* or *SDC1* results in a complete loss of H3K4me3, a significant decrease of H3K4me2 but no change in H3K4me1, while inactivation of *SPP1* or mutating *SWD2* (*swd2K68,69R*) results in a severe and specific decrease of H3K4me3 (Figure 1A).

The same strains were analyzed in parallel by long oligo DNA microarray expression-profiling, targeting the coding strand of virtually all yeast genes. Throughout this study all microarray analyses were performed with four replicates (two independent cultures, each measured in duplicate, Materials and Methods). In addition, controls were included that allow detection of global changes in the entire mRNA population [41]. Such global changes were not detected. In agreement with the most recent studies of *SET1* deletion on its own [29] [30], expression of only a minority of genes is affected in the different COMPASS mutants. Within the entire set of deletion mutants, 89 genes changed significantly in at least two mutants (p-value lower than 0.01 and fold-change versus wild-type more than 1.7), with 69 genes showing increased expression and only 20 exhibiting decrease (Figure 1B). Deletion of any of the five core subunits Set1, Swd1, Swd3, Bre2 and Sdc1 leads essentially to the same expression profile (Figure 1B and Figure S1).

It is interesting to compare the changes in gene expression to the H3K4 methylation states observed in the different mutants. Virtually no significant changes in gene expression are observed in *spp1*Δ or in the *swd2*K68,69R mutant (Figure 1B) that both show a specific and severe decrease of H3K4 trimethylation (Figure 1A). Changes in gene expression are observed in *bre2*Δ and *sdc1*Δ, where H3K4 dimethylation is significantly diminished on top of the loss of trimethylation, (Figure 1A, 1B). The additional loss of H3K4 monomethylation, as observed in *set1*Δ, *swd1*Δ or *swd3*Δ (Figure 1A), does not lead to additional changes in gene expression (Figure 1B). Because of the correlation between their location and transcription rates [25], H3K4 methylation marks in yeast have generally been associated with transcription activation. The main effect of mutating COMPASS components in *S. cerevisiae* is nevertheless derepression (Figure 1B). Furthermore, the effect is only strong upon loss of dimethylation on top of trimethylation loss, which on its own has little effect.

To distinguish whether the repressive effect of COMPASS is related to H3K4 methylation or is due to an unidentified methylation target of Set1, a H3K4 point mutant was analyzed. The predominant effect is up-regulation (Figure 1C) and the overlap with the COMPASS-repressed genes is highly significant (p-value 3.1*10^−27^, hypergeometric test). An apparently lower number of genes is derepressed in the H3K4 point mutant. As analyzed later, this is likely related to the H3/H4 histone dosage effect of the strain used to generate the point mutant. To nevertheless investigate the possibility that Set1 repression is mediated by a target other than H3K4, *SET1* was deleted in the H3K4 point mutant strain. DNA microarray analysis of the double mutant shows a completely epistatic relationship with no additional effect of deleting *SET1* in the H3K4 point mutant strain (Figure S2). This confirms that the repressive effect of COMPASS observed here is mediated through H3K4.

It has been previously shown that H3K4 di- and tri-, but not monomethylation states are controlled by the Rad6/Bre1-mediated monoubiquitylation of histone H2BK123 via a trans-tail pathway involving ubiquitylation of Swd2 [23], [42], [43], [44], [45]. To investigate whether the repressive effects of H3K4 methylation are mediated by this pathway, a *bre1*Δ strain was analyzed. Changes in gene expression in *bre1*Δ matches the COMPASS mutants profiles with a highly significant overlap (p-value of 1.0*10^−37^, hypergeometric test) (Figure 1C). The repressive effects observed here therefore correspond to the action of the entire pathway starting from ubiquitylation of histone H2B and leading to di- and trimethylation of H3K4.

### Repression dynamics are subtly affected by loss of H3K4 methylation

**Figure 2:**
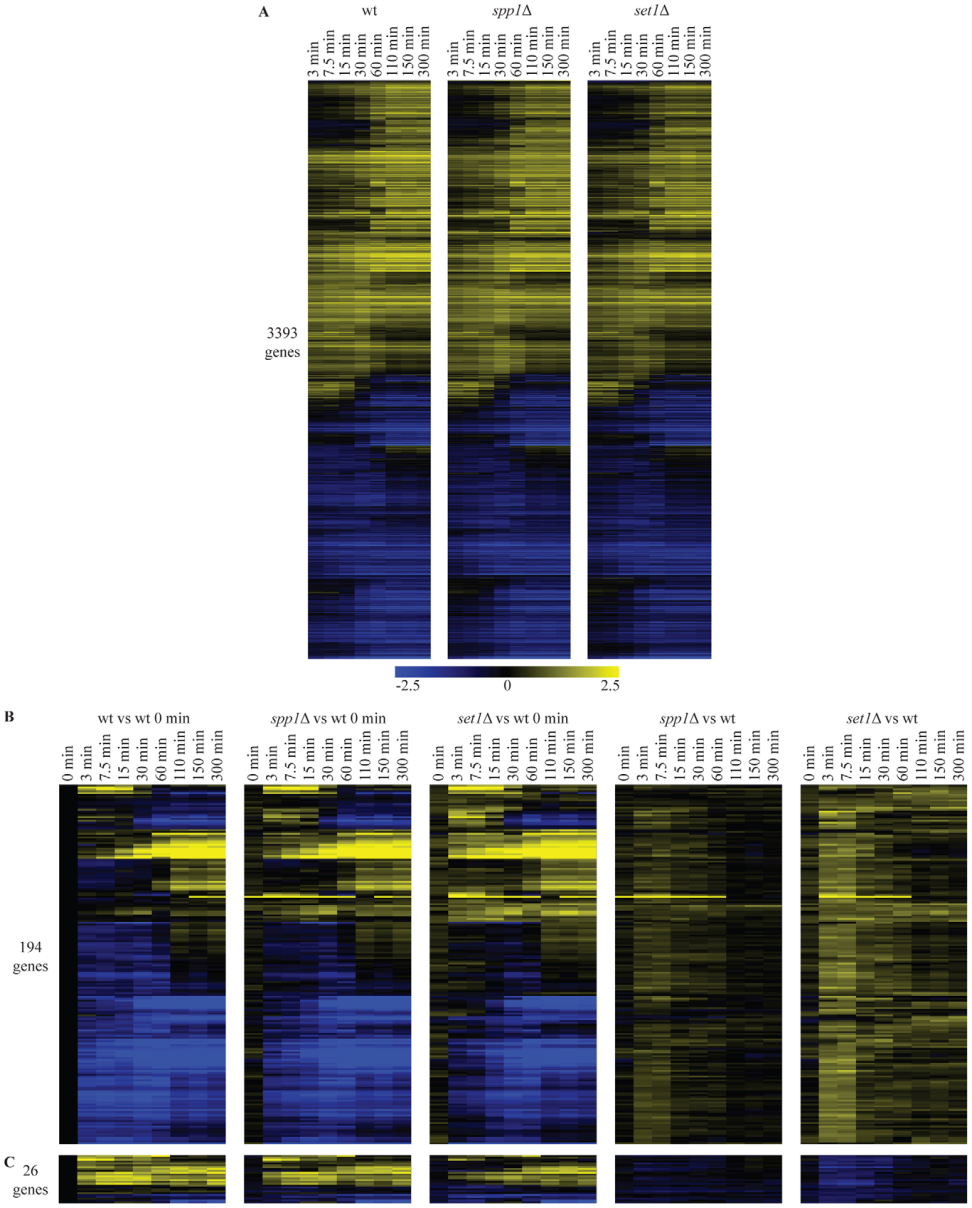
Loss of H3K4 di- and trimethylation leads to a delay in repression kinetics for a subset of genes. (A) Hierarchical clustering of all genes with significant changes in mRNA expression during the shift from low to high glucose in any of the wt, *spp1*Δ and *set1*Δ time-courses. The log_2_ values correspond to the difference with the zero time point of each time-course. (B) Hierarchical clustering of genes with delayed repression compared to wt. These genes were identified based on statistically significant differences between the mutant and wt time-courses (Materials and Methods). The first three panels show the differences in expression versus the wt zero time point. The last two panels (*spp1*Δ *vs wt*) and (*set1*Δ *vs wt*) depict the differences between mutant and wt for each different time point, by subtracting the log base 2 gene expression ratios of the wt time-course from the mutant time-course. (C) Hierarchical clustering of genes that show delay in activation.

Since the experiments described above deal with steady-state changes in mRNA levels, we next asked whether the absence of H3K4 methylation would affect the kinetics of gene expression changes. This is based on the proposal that H3K4me3 may have a memory function, bookmarking genes that require rapid induction under specific growth conditions, both in mammals [46] and yeast [24]. For this purpose, wild-type (wt), *set1*Δ (absence of all three H3K4 methylation states) and *spp1*Δ (lack of H3K4 trimethylation only) were expression-profiled at multiple time-points during the transition from post-diauxic shift to early log phase, a transition during which a large number of genes change expression levels [47]. During this transition, expression of approximately 3400 genes change significantly in wt cells, covering a broad range of gene expression dynamics (Figure 2A). No major differences in the transcription kinetics between wt and the two mutant strains are observed. This indicates that disruption of H3K4 methylation or H3K4 trimethylation on its own does not have a global effect on the dynamics of transcription (Figure 2A), even though most active genes exhibit H3K4me3 marks [6], [11], [27]. These results also agree with the lack of a global effect after removing the H3K4me3 mark under steady-state conditions (Figure 1B).

A detailed statistical analysis for genes showing differences in their induction or repression kinetics in the mutants was also performed among the 3400 genes that change significantly during the time-course experiment. In the *set1*Δ (loss of all three H3K4 methylation states) time-course, 220 genes show statistically significant differences in their expression kinetics compared to the corresponding time points in wt (compare Figure 2B, 2C first and third panel). The vast majority of these (194 genes - Figure 2B) exhibit defective repression, observed as delayed repression or faster activation. Only a minority of genes exhibit an activation defect (26 genes - Figure 2C). To facilitate visualization of these mostly quite subtle changes, the wt time-course was subtracted from each mutant time-course. This results in the right-hand panels of Figure 2B and 2C, showing for each time-point, the difference in expression levels for each mutant relative to the wt at the same time point. For *spp1*Δ (loss of H3K4me3), only 15 genes exhibit any differences in their expression kinetics (Figure 2B, 2C, second panel). These all belong to the 220 genes with slightly altered kinetics in the *set1*Δ time-course. In agreement with the steady-state analysis, the effects detected in the time-course experiments are thus virtually all attributable to the complete loss of methylation observed in *set1*Δ, rather than to the specific loss of H3K4me3 observed in *spp1*Δ. The results concur with a repressive role for COMPASS on mRNA expression of a subset of genes, as observed in the steady-state experiments too (Figure 1) with an extremely significant overlap between the affected genes (p-value 6*10^−35^), as expected.

### Unconventional methylation patterns at the 3′-end of COMPASS-repressed genes

We next investigated whether there are any particular characteristics shared by the set of genes upregulated upon mutation of COMPASS components (Figure 1B). In agreement with a recent analysis of *set1*Δ [29], statistically significant enrichment for location close to telomeres is observed (Figure S3). Among the 69 COMPASS-repressed genes, 10 are telomere-proximal (within 15 kb) Figure S3 and Table S1). Although this enrichment is significant, in most cases the expression of adjacent genes was not found to be affected by the deletion of COMPASS subunits. For instance, *PHO11*, *SNO4*, *MCH2*, *SOR2*, *YGL258W-A* and *PHO12*, that are located between 4 and 10 kb from the telomeric DNA on different chromosomes (Table S1) are all flanked by genes that are not affected by the absence of Set1. This, as well as the small number of all telomere- proximal genes being derepressed in the COMPASS mutants makes it unlikely that the observed derepression of telomere-proximal genes is only caused by loss of the Sir-dependent telomeric position effect [19], [48], [49], [50], [51], [52].

**Figure 3:**
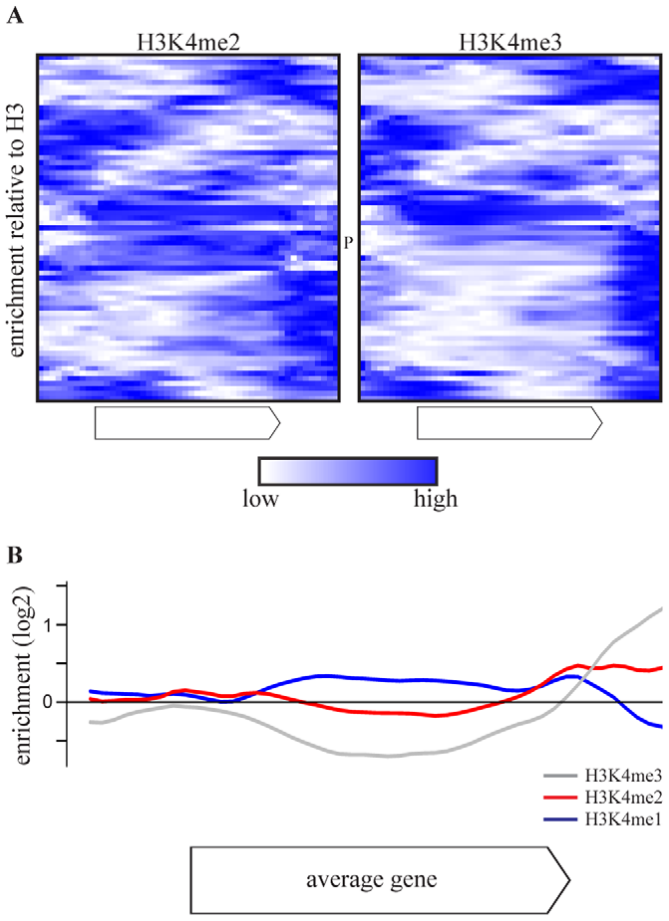
COMPASS-repressed genes have aberrant H3K4 methylation patterns, indicative of 3′-end antisense transcription. (A) Heatmaps of enrichment of H3K4me2(left) and H3K4me3(right) over H3 in the gene body and flanking regions of the 69 COMPASS repressed genes, based on [53]. The enrichments are rescaled for each individual gene with blue and white corresponding to the highest and lowest enrichment, respectively. *PHO84* is marked by P. (B) The average enrichment of H3K4me1 (blue), H3K4me2 (red) and H3K4me3 (grey) over H3, for the set of 47 COMPASS-repressed genes that show at least a two-fold enrichment of H3K4me2 or H3K4me3 somewhere across the gene or flanking region [53].

As the effect of COMPASS deletions is attributable to H3K4 methylation (Figure 1C), the H3K4 methylation patterns of COMPASS-repressed genes were examined using chromatin immunoprecipitation data from a wt strain from the same genetic background, grown under similar conditions [53]. Intriguingly, the di- and trimethylation patterns of the 69 COMPASS-repressed genes (Figure 3A) deviate from the average gene which has enrichment of H3K4me3 around the transcription start site (Figure S4) [25], [53]. Instead, the majority of COMPASS-repressed genes show enrichment of H3K4me3 at the 3′-end or in the body of the gene. In the minority of cases where 5′-end enrichment is observed, this is accompanied by a second trimethylation peak at the 3′ end. To exclude that the deviating localization of peaks is not due to measurement noise or signal originating from neighbouring genes, the methylation profiles are averaged in Figure 3B only for those genes that have a greater than 2-fold enrichment of H3K4 methylation on any portion of the gene. This average pattern for COMPASS-repressed genes shows a clear enrichment of H3K4me3 at the 3′ end, followed by H3K4me2 enrichment in the gene body, which is in turn followed by H3K4me1 further towards the 5′-end. Genes repressed by COMPASS therefore show abberant H3K4 methylation patterns that are characterized by a reversed orientation of the normal H3K4 methylation pattern observed for active genes [3].

### Promotion of 3′-end antisense transcription by Set1 contributes to repression on coding genes through two distinct mechanisms

A plausible explanation for the H3K4 di- and trimethylation peaks at the 3′-ends of COMPASS-repressed genes is the presence of antisense transcription initiation at the 3′-end of the coding region, leading to non-coding transcription over the same genomic location but in the opposite direction of the sense transcription. The DNA microarrays used in the previous experiments are coding strand-specific and do not detect anti-sense transcripts. However, two recent genome-wide surveys of non-coding transcripts [39], [40], do detect antisense RNAs for more than 85% of the COMPASS-repressed genes (Table S2). Interestingly, *PHO84* belongs to the group of COMPASS-repressed genes identified here (Figure 3A, marked with P) and has been shown to be regulated by antisense RNA transcripts originating from its 3′-end both *in cis* and *in trans* [32], [34]. We therefore investigated the manner in which 3′-end antisense transcription may be involved in Set1-mediated repression.

**Figure 4:**
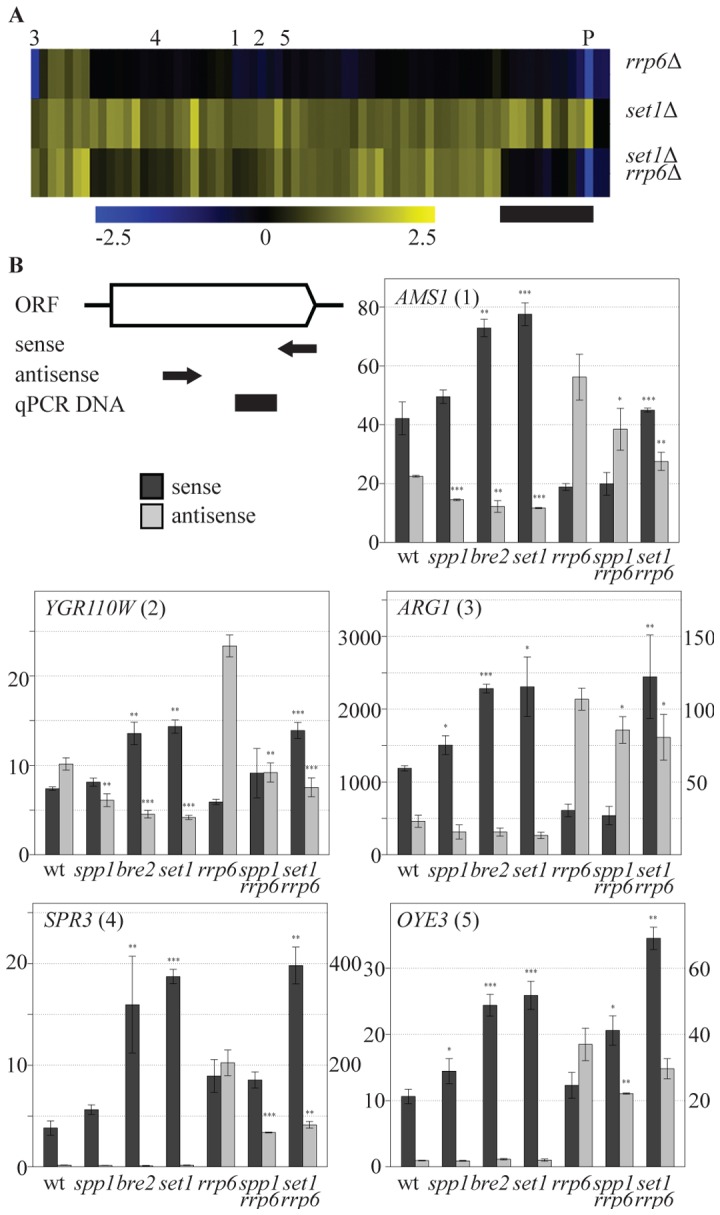
COMPASS repression is mediated through 3′-end antisense transcriptional gene silencing. (A) Hierarchical clustering of the 69 COMPASS-repressed genes in the *rrp6*Δ, *set1*Δ and the *set1*Δ *rrp6*Δ strains. *PHO84* is marked by P and *AMS1*, *YGR110W*, *ARG1*, *SPR3* and *OYE3* are marked by 1 to 5, respectively. The black bar marks the subset of genes where the two mutations are epistatic. Three quarters of these genes are related to phosphate metabolism. (B) Sense and antisense RNA levels analyzed by qPCR in indicated backgrounds. The schematic representation of the follow-up genes shows the relative positions of the primers used for strand-specific reverse transcription reactions in arrows, while the black box indicates the location of the DNA fragment produced during the qPCR. Error bars reflect standard deviations of an average signal obtained from at least two independent experiments. The significance of the difference in expression changes observed between the mutant cells and the corresponding background strain (rrp6*Δ* in the case of *spp1Δrrp6Δ* and *set1Δrrp6Δ*, *wt for the others*), was evaluated using Student's *t*-test (^*^ *P* 0.01–0.05; ^**^ *P* 0.001–0.01; ^***^ *P*<0.001).

One hallmark of the mechanism of repression of *PHO84* is the contribution of the antisense transcript itself rather than only the process of antisense transcription. Stabilization of the antisense transcript by deletion of the exosome component *RRP6* [32] is sufficient to repress sense *PHO84* transcription. To test whether COMPASS repression is mediated by 3′-end antisense transcripts, an *rrp6*Δ profile was generated and compared to *set1*Δ. Deletion of *RRP6* affects expression of 117 coding genes in total (p<0.01, fold-change>1.7) and does not have a general effect on all COMPASS-repressed genes (Figure 4A). In agreement with previous studies however, a significant down-regulation of *PHO84* is observed (marked P in Figure 4A). Lack of down-regulation of the other COMPASS-repressed genes in *rrp6*Δ may be simply due to an already repressed state in wt. Since these genes are derepressed in *set1*Δ, the possible involvement of antisense transcripts in repressing all COMPASS-affected genes was further tested by analysis of an *rrp6Δ set1*Δ double mutant (Figure 4A). The double mutant expression-profile reveals two classes of COMPASS-repressed genes. On the smaller group of genes (Figure 4A, marked with a black bar), that includes *PHO84* as well as several other phosphate-related genes, an epistatic effect is observed in *rrp6Δ set1*Δ, whereby the upregulation in *set1*Δ is lost in the double mutant. This implies that the antisense transcript mediated repression of sense genes is not unique for *PHO84*, but is shared with functionally related genes. Such genes are the exception however. The largest group of Set1-repressed genes behaves in a different manner, still showing derepression in the double mutant, similar to their behaviour upon deletion of *SET1* on its own. This therefore likely represents a distinct mechanism of COMPASS repression.

In order to understand the mechanism by which COMPASS represses coding transcription in an exosome-independent manner, five representative genes from this group, *AMS1*, *YGR110W*, *ARG1*, *SPR3* and *OYE3* (indicated by 1 to 5 in Figure 4A), were analyzed in greater detail. These genes represent different functional categories, different telomeric proximities and different types of antisense transcripts, as suggested by the genome-wide datasets. The first three genes contain antisense stable unannotated transcripts (SUTs), while the other two have antisense cryptic unstable transcripts (CUTs) [40]. The location of H3K4 methylation patterns [53] corresponds to the location of the transcription initiation sites of these antisense transcription units (Figure S5). The effects of different COMPASS mutants on both sense and antisense transcription of these genes were analyzed by quantitative RT-PCR using strand-specific primers (Figure 4B).

Sense transcript upregulation of the five genes is observed in *set1*Δ (Figure 4B), that exhibits loss of all H3K4 methylation marks (Figure 1A), in *bre2*Δ (Figure 4B), that exhibits loss of all H3K4me3 and most H3K4me2 (Figure 1A) and in *set1*Δ combined with *rrp6*Δ (Figure 4B), all in agreement with the sense-specific microarray results (Figure 1B, Figure 4A). In *bre2*Δ and *set1*Δ, upregulation of sense transcription is accompanied by a decrease in antisense transcription (Figure 4B, panels 1–3). As expected, changes in antisense CUT transcription are not evident without prior stabilization by the exosome deletion (Figure 4B, panels 4,5). For all five genes, stabilisation of antisense transcripts does occur in *rrp6*Δ (Figure 4B), but these increased antisense levels do not necessarily result in more repression of sense transcription (Figure 4B) as is clearly the case for *PHO84* ([32] and Figure 4A). This confirms that an increase in antisense transcript levels through *rrp6*Δ-dependent stabilisation is not the mechanism of COMPASS repression for these genes. Rather, the data suggest that it is the process of antisense transcription itself that represses the sense transcription.

### The Set1 repressive effect is mediated through 3′-end antisense transcription

**Figure 5:**
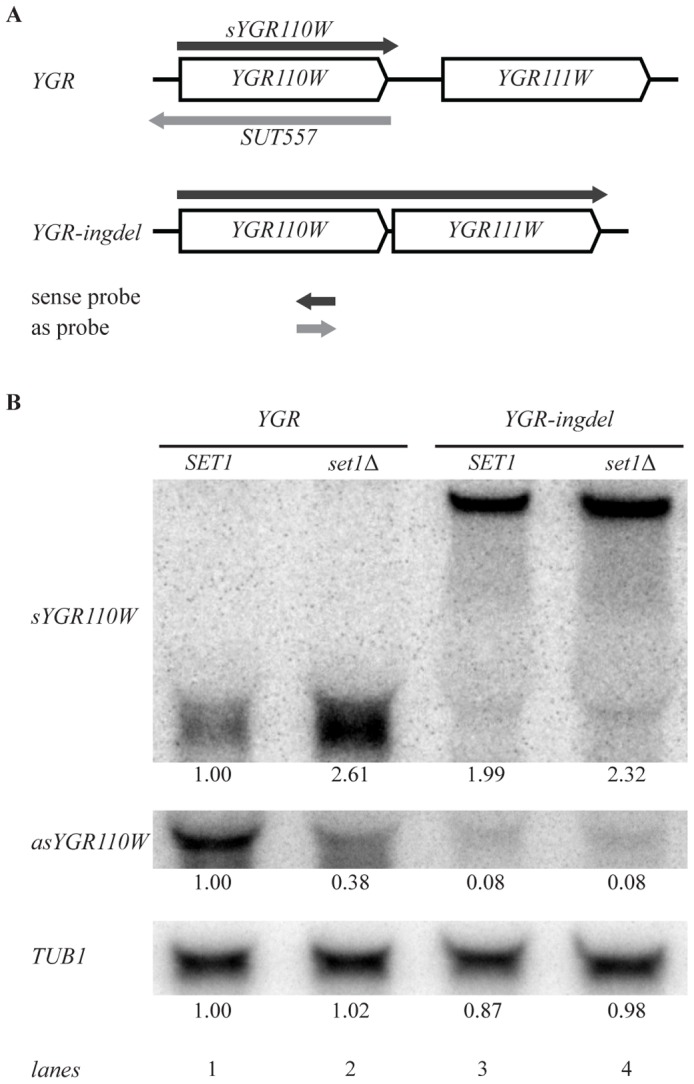
Set1 represses sense transcription through promotion of antisense transcription. (A) Scheme showing *YGR110W* and *YGR111W* genes before (YGR) and after (YGR-ingdel) deletion of their intergenic region. The sense *YGR110W* transcript (*sYGR110W*), as well as the longer sense transcript in the YGR-ingdel strain are shown in dark grey, while the antisense transcript (SUT557) is shown in light grey. The position and 5′-3′ direction of the strand-specific probes used to detect the transcripts are also shown. (B) Autoradiographs of Northern blots hybridized with the strand-specific DNA probes designed to detect the sense (*sYGR110W*) or antisense (*asYGR110W*) transcripts of *YGR110W* in the YGR and YGR-ingdel strains with wild-type (*SET1*) or deleted *SET1* (*set1Δ*). An autoradiograph of the same blot hybridized with a tubulin probe (*TUB1*) was used as loading control. Quantitation of the bands are shown below each panel relative to the wt (SET1 YGR) strain for TUB1 and relative to the wt (SET1 YGR) strain and the loading control for the *sYGR110W* and *asYGR110W* panels.

Because sense and 3′end antisense transcription seem coupled [54], it is difficult to distinguish whether the increased sense transcription in COMPASS mutants is caused, or is followed, by a decrease in 3′-end antisense transcription. One way of addressing this directly is to eliminate 3′-end antisense transcription by other means than through disruption of COMPASS. For this purpose strong terminator sequences were introduced downstream of the five model genes analyzed in Figure 4B, either as insertions between antisense promoters and the end of the ORF, or as replacement of complete intergenic sequences. Neither approach resulted in loss of 3′-end antisense transcription, which agrees with the recent finding that terminators can function as promoters [55] . Disruption of 3′-end antisense transcription was then attempted by removal of all, or a significant part of the intergenic region. Complete loss of all antisense transcription was only observed for the YGR110W intergenic deletion mutant, which we further analyzed in depth (YGR110W-ingdel, Figure 5).

Strand-specific Northern blot analysis of YGR110W-ingdel shows that loss of antisense transcription (Figure 5B, asYGR110W, lane 1 versus lane 3), is accompanied by derepression of sense transcription (Figure 5B, sYGR110W). This demonstrates that 3′-end antisense transcription results in repression of sense transcription. Furthermore, introduction of *SET1* deletion into the YGR110W-ingdel strain, does not result in significant further derepression as is observed in the presence of 3′-end antisense transcription (Figure 5B, lanes 1 and 2 versus lanes 3 and 4). This agrees with the proposal that the repressive effect of COMPASS on coding genes is a result of promoting 3′-end antisense transcription.

### H3K4me3 promotes 3′-end antisense transcription and H3K4me2 contributes to coding gene repression

The results presented in Figure 4B and Figure 5B imply a positive role for Set1 on antisense transcription. *SET1* deletion results in loss of H3K4me1, me2 and me3 (Figure 1A). *SPP1* deletion (loss of H3K4me3 only), has little effect on sense transcript levels (Figure 1, Figure 2, Figure 4B). *SPP1* deletion does result in decreased antisense transcripts as observed either in the presence or absence of *RRP6* (Figure 4B). Our results indicate that H3K4 trimethylation, which is found at the 3′-end of these genes, has a role in promoting 3′-end antisense transcription. This effect is not absolute however. Antisense transcripts are reduced in the *SET1 RRP6* double deletion compared to *rrp6*Δ, but are not completely absent. This indicates that antisense transcription is promoted by, but not fully dependent on, H3K4me3. Since *spp1*Δ still exhibits wt levels of H3K4me2 (Figure 1A) and virtually no derepression of sense transcription (Figure 1B and Figure 4B), this indicates that it is the H3K4me2 mark which is most important for repression of sense transcription on these genes. Together, the results of these experiments are consistent with a model, whereby the majority of COMPASS-repressed genes are maintained in an inactive state through 3′-end antisense transcription that is in part promoted through H3K4me3 at the 3′-end, and in turn deposits a repressive H3K4me2 mark further into the body of the gene.

### COMPASS repression is mimicked by reducing nucleosome levels

We next asked what determines the specificity of the effects observed upon mutation of COMPASS. H3K4 methylation marks all active genes and approximately one third of all genes exhibit antisense transcripts [40], yet only a subset are affected by deleting COMPASS subunits (Figure 1). It has recently been proposed that the transcription factor Reb1 may drive non-coding transcription, either from neighbouring genes [33] or from the promoter of the antisense transcript itself [56]. Reb1 binding sites are found downstream of only three of the 69 COMPASS-repressed genes. There is also no statistically significant enrichment for Reb1 binding sites in the ORFs or flanking regions of genes up-regulated in the COMPASS mutants. Both observations suggest that the specificity of Set1 repressive effects is not generally linked to Reb1. In addition, no other putative regulatory motifs could be detected in these regions using different search algorithms [57].

**Figure 6:**
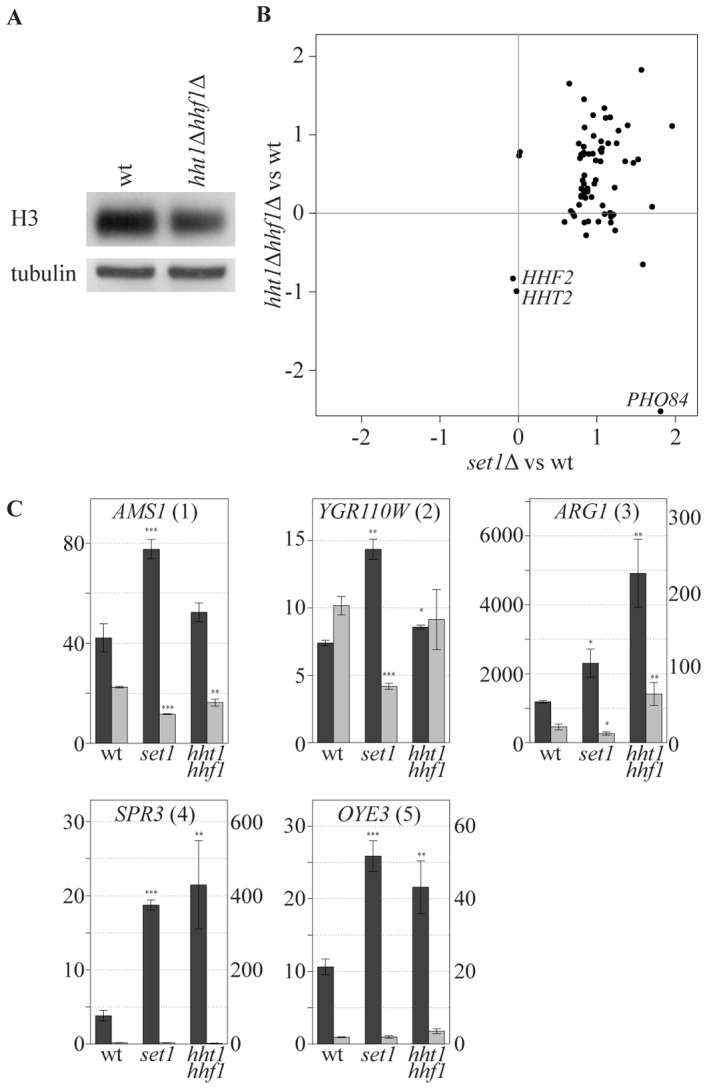
COMPASS-repressed genes are derepressed upon decrease in nucleosome content. (A) The cellular expression level of H3 was analyzed by Western blotting in wt and *hht1*Δ *hhf1*Δ cells. (B) Correlation of the effects, as measured by log base 2 ratios, of low nucleosomes levels (*hht1*Δ *hhf1*Δ) and *SET1* deletion (*set1*Δ) on the COMPASS-repressed genes versus wt cells. *LYS2* is excluded as it is used as an auxotrophic marker for the *hht1*Δ *hhf1*Δ strain. *PHO84* is marked, as it is significantly repressed in the low nucleosome strain. The two histone genes are also depicted verifying the expected 2-fold reduction in their mRNA levels. (C) Strand-specific qPCR analysis, as in Figure 4B, for the indicated backgrounds.

An alternative explanation for the specificity of COMPASS repressive effects is that specificity is dictated by increased sensitivity of specific genes to a particular chromatin structure which is influenced by H3K4 methylation. While profiling strains with altered histone expression levels we noted an interesting correlation with the collection of COMPASS mutants. To investigate this, a strain bearing single copies of the histone H3 and H4 genes under control of their native promoters [58] was analyzed. The two-fold reduction in mRNA levels of H3 and H4 in this strain (Figure 6B, marked *HHT2* and *HHF2*) is accompanied by slightly decreased H3 and H4 protein levels (Figure 6A). Interestingly, this results in upregulation of a specific subset of genes that strongly correspond to the genes upregulated upon *SET1* deletion (p-value 1.4*10^−23^, Figure 6B). Although the overlap is highly statistically significant, it is not complete and does not extend to *PHO84* for example, in agreement with the proposal for a distinct repressive mechanism for such genes (Figure 4A). *SET1* deletion does not globally affect nucleosome levels (data not shown), and antisense transcript levels are not reduced in the single copy H3 H4 strain (Figure 6C). Besides antisense transcription, a second common property of the genes affected by loss of COMPASS function is therefore sensitivity to histone abundance. Since histone abundance affects nucleosome density [59], this suggests that Set1 may repress genes by effecting nucleosome density. As is discussed below, one manner in which this may be achieved is through the repressive H3K4me2 mark that is laid down through 3′-end antisense transcription.

## Discussion

### Repressive role of COMPASS in *S. cerevisiae*

The results presented here add to a number of reports that indicate that the major non-redundant role of COMPASS in *S. cerevisiae* is repression of coding genes [34], [56]. Early genome-wide analyses of *set1*Δ yielded conflicting results, in two cases pointing to global positive effects [27], [28] and in one case ignoring the prevalence of specific repressive effects [6]. Some of the differences between these studies and the current one can in retrospect be attributed to use of double-stranded cDNA arrays, less convenient for discriminating between sense and ant-sense effects, as well as to normalisation issues. The analyses presented here, using strand-specific techniques, with replicate experiments for a variety of different mutants under both steady-state and dynamic conditions, indicates that removal of H3K4me3, a global mark of active transcription, has no global effect. The repressive effects observed on a specific subset of genes agree with the most recent other genome-wide analyses of *set1*Δ [29] [30], as well as with the fact that deletion of *SET1* is not lethal. Gene Ontology analysis of the affected genes reveals an overrepresentation of vitamin metabolism (essentially thiamin biosynthesis) and spore wall assembly (Table S3) in agreement with the cell wall and stationary phase defects previously observed in *set1*Δ cells [51].

### COMPASS and antisense transcription

What is the mechanism of the observed repression? Despite the fact that COMPASS-repressed genes show a significant enrichment for telomeric-proximal localization (Figure S3 and [29]), Set1-dependent repression of these genes due to a telomere position effect can probably be ruled out since the derepression observed in *set1*Δ only affects a small percentage of individual genes within these regions. Only a few of the affected genes are close to telomeres and only few telomere-proximal genes are affected. Analysis of methylation patterns (Figure 3 and [53]), non-coding RNA maps (Table S2 and [39], [40]) and the comparison of mutants with different methylation states support a model whereby COMPASS mediates repression of coding genes by promoting the expression of 3′-end antisense transcripts through deposition of H3K4me3 at their 3′-end.

An involvement of Set1 in promotion of 3′-end antisense transcription, resulting in a repressive effect on sense transcription has been reported for *PHO84*, which is repressed through the presence of antisense transcripts [34]. Our results are consistent with a repressive role for Set1 on *PHO84*. The genome-wide nature of our experiments indicates however that the majority of Set1 affected genes are repressed through a different mechanism, independent of the level of antisense RNA transcripts. Rather, for the majority of Set1-regulated genes, repression is caused by the process of antisense transcription itself. This mechanism is therefore related to the recently reported attenuation in *GAL10-GAL1* activation which is also facilitated through cryptic transcription [56]. One major difference is that for the mechanism reported here, COMPASS is required to maintain antisense transcription whereas this does not seem to be the case for the cryptic transcription observed at the *GAL10-GAL1* locus [56].

### Distinct roles for H3K4me2 and H3K4me3

The comparison of different COMPASS mutations carried out here, facilitates distinguishing between the roles of the different H3K4 methylation states. Mutants with grossly lowered or completely absent H3K4me3 exhibit decreased antisense transcription. However this only results in derepression of the coding gene if H3K4me2 is also abolished. The positive role of H3K4me3 on antisense transcription fits with the correlation observed between the presence of this mark and promoter activity of coding genes [7], [8], [9], [10], [11], [25]. Most non-coding RNAs originate from nucleosome-free regions (NFRs) shared with protein-coding transcripts [40]. This is also the case for three of the five COMPASS-repressed genes analyzed here in detail (*AMS1*, *ARG1* and *OYE3*). Interestingly, despite sharing a NFR with the downstream protein-coding gene, loss of H3K4 trimethylation causes reduction of antisense transcription without affecting transcription of the flanking protein-coding gene in each case. This fits with the observation that bidirectional transcription from a single NFR, originates from two distinct preinitiation complex recruitment sites [60]. This may indicate the presence of redundant mechanisms for maintaining protein-coding gene transcription in the absence of H3K4me3, which are lacking for the antisense non-coding transcription originating from the same NFR. Another, non mutually exclusive mechanism, can be that lack of H3K4 trimethylation in the antisense transcription start site increases the recruitment of corepressor complexes, such as Rpd3S [61], that repress the expression of the non-coding transcript, but not that of the coding gene [62].

The results also indicate a role for the H3K4me2 mark in facilitating repression. This agrees with several recent studies suggesting mechanisms through which H3K4me2 may play a repressive role. For example, it has recently been reported that H3K4me2 in the body of active genes is recognized by the Set3 complex, leading to histone deacetylation, a repressive chromatin state [63]. A different histone deacetylase, Rpd3, has been implicated in the repressive role involving Set1 on the GAL10-GAL1 locus [56]. Furthermore, methylation of H3K4 protects against an H3 tail endopeptidase recently described in *S. cerevisiae* and humans that facilitates transcription initiation and precedes histone eviction [64], [65]. All these possible mechanisms fit with the observation made here that globally reducing H3 and H4 levels mimics the derepression of COMPASS mutants. The degree of overlap between the COMPASS mutants' profiles and the histone depletion profile also give an explanation for the specificity of the COMPASS repression. Genes repressed by COMPASS have antisense transcription, but are also sensitive to nucleosome density.

Sensitivity to histone depletion may not be the only reason for the lack of genome-wide effects upon COMPASS mutation. Functional redundancy may also contribute. One of the prevalent ideas for a general role of H3K4 methylation in *S. cerevisiae* is that transcription-associated H3K4 methylation, as well as deposition of the histone variant H2A.Z, antagonizes the local spread of Sir-dependent silent chromatin into adjacent euchromatic regions [52], [66], [67]. It has recently been shown that H2A.Z deposition and Set1 cooperate to prevent Sir-dependent repression of a large number of genes located across the genome [29]. This functional redundancy between H3K4 methylation and H2AZ deposition may thus buffer transcription from changes in euchromatin, thereby minimizing the observed effects of H3K4 methylation loss.

This work offers a plausible explanation for how a transcription factor, previously thought to positively contribute to transcription, can nevertheless exert a negative effect, through promoting antisense transcription. The opposite has previously been shown for regulation of *IME4*. Here a repressor complex binds to the promoter of an antisense transcription unit in the 5′-end of *IME4* and by repressing the antisense transcription, facilitates the *in cis* sense transcription activation [68]. Although further work is required to pinpoint the mechanisms further downstream of H3K4 di- and trimethylation, COMPASS exemplifies the growing insight that the roles of histone modifications in gene expression are non-linear [69] and context-dependent [70].

## Materials and Methods

Microarray data is accessible through the public microarray database ArrayExpress (http://www.ebi.ac.uk/arrayexpress/) under accession number E-TABM-486. The accession numbers below refer to detailed protocols in ArrayExpress.

### Strains and primers

Strains and primers used in this study are described in Tables S4 and S5 respectively. The YGR110W-ingdel strain was created by first inserting a cassette containing the *Sp HIS5* gene in reverse orientation flanked by the *Ag TEF* promoter, terminator sequences and loxP sites from plasmid pUG27 [71] to replace the intergenic region between *YGR110W* and *YGR111W* using YGR110W_HIS5_F and YGR110W_HIS5_R primers. Subsequently the cassette was floxed out by transforming the strain with the plasmid pSH47 and expressing Cre recombinase as previously described [72].

### Histone purification and Western blotting

Histones were purified as described [42], subjected to 16% SDS-polyacrylamide gel electrophoresis, and either Coomassie Blue stained or transferred to 0.2 µm Protran^R^ nitrocellulose. Antibodies used to detect mono-, di- and trimethylated H3K4 and histone H3 were from Abcam.

### Cultures

Two independent colonies of each strain were first inoculated and grown overnight in synthetic complete medium with 2% glucose. For the mid-log/steady-state experiment, larger cultures were inoculated the next day at an OD600 of 0.15 in fresh medium, allowed to grow at 30°C and harvested at OD600 0.6, (P-UMCU-36). For the time-course experiment, overnight cultures were used to inoculate 50 ml cultures at an OD600 of 0.15. These were allowed to deplete glucose by growing for 24 hours and were used the next day to start 500 ml cultures at an OD600 of 0.15 in fresh medium for the time-course sampling (P-UMCU-47).

### RNA isolation and amplification

Total RNA isolation was by hot acid phenol (P-UMCU-37) and cleaned up using RNeasy (Qiagen). Before amplification, external RNA controls were added to total RNA to check for global shifts in mRNA levels [41]. cRNA amplification and labelling using amino-allyl UTP was performed on a Caliper robot system (P-UMCU-38).

### Microarrays and hybridizations

Each sample was generated twice, as independent biological replicates. These were hybridized in dye-swap against a common wt reference RNA (P-UMCU-39) on oligo-arrays that represented each gene twice (P-UMCU-34). After scanning (P-UMCU-40), raw data were extracted with Imagene (Biodiscovery) (P-UMCU-42).

### Data analysis

Since spike-in of external RNA controls revealed no global changes in the mRNA population [41] for the mid-log experiment, non-background corrected data were normalized with print-tip LOESS [73] on gene probes with a span of 0.4 (P-UMCU-41). For the time-course, all features, including negative and external controls (EC) except EC 4, 6 and 8, were used for the estimation of the LOESS curve (P-UMCU-46). Probes flagged as absent, or with a nearly saturated signal were not used to estimate the LOESS curve. For differential expression analysis, the LIMMA package [74] was used. Mitochondrial-encoded genes and Ty elements were excluded due to their high biological variation. Genes with an FDR-adjusted p-value less than 0.01 and a fold-change of more than 1.7 were considered significant. These thresholds are based on systematic analyses of the variation observed in a collection of more than 100 wt expression profiles [75]. For the time-course experiment, changes were considered significant if they fulfilled these criteria for two consecutive time-points. Hierarchical clustering was by MeV [76], using standard correlation and average linkage. Analysis of overlap between genelists of significantly changing genes of two expression profiles was by hypergeometric test. For the GO and transcription factor enrichment analysis, a right-sided Fisher's exact test was used and the p-values were corrected for multiple testing using Bonferroni. The GO annotations were obtained from SGD.

For the H3K4 methylation ChIP-chip analysis, the data are from [53]. For each gene, a region corresponding to the ORF plus 500 bps in both directions was used. The ORF was divided into 30 bins of equal length and the flanking regions in 10 bins each. A loess algorithm [77] with a span of 0.2 was used to estimate the enrichment of the methylation marks for every bin.

### Reverse Transcription and qPCR

cDNAs of sense RNA or antisense RNA were generated by SuperScript III Reverse transcriptase (Invitrogen) from total RNAs using gene and strand-specific primers. For each gene, cDNAs obtained from the reverse transcription of sense or antisense RNA were quantified by a real-time qPCR with gene-specific primers corresponding to a 150 bp fragment (Figure 4B). The same primers were used to quantify sense and antisense cDNA of each gene. The position and the sequence of each primer are indicated in Figure 4B and Table S5.

### Northern blotting

The strand-specific DNA probes used to detect the presence of sense and antisense transcripts of *YGR110W* are shown schematically in Figure 5A. First a cold PCR product template was obtained using primers 3′qYGR110W and 5′qYGR110W. Subsequently the hot ssDNA probes for detection of the sense and antisense transcripts were generated from the template using the first or the second primer, respectively, in linear PCR reactions. Quantitation of the radioactive signal was performed using ImageQuant (Molecular Dynamics).

## Supporting Information

Figure S1 (A) Hierarchical clustering, as in Figure 1B, of all genes with significantly changed mRNA expression in any COMPASS mutant. Figure 1B depicts those genes that have significantly changed expression in at least two mutants. (B) Genes depicted in the same order as in A for the H3K4R point mutant and *bre1*Δ.
(TIF)

Figure S2 The repressive effect of Set1 on transcription is through H3K4 (A) Gene expression scatter plot of the average, normalized fluorescent intensity values of each gene in *set1Δ* compared to the wt strain. The 69 COMPASS-repressed genes are represented by yellow dots. (B) As in A, but now for the *set1Δ* H3K4R double mutant compared to the H3K4R point mutant. The deleted gene is represented by a blue dot.
(TIF)

Figure S3 COMPASS-repressed genes are enriched near telomeres. The histogram shows the genomic location of the 69 genes significantly upregulated in at least two COMPASS deletion mutants. The bars represent the numbers of genes found in 5-kb intervals from nearest chromosome end. The line represents the log_10_ p-value as a function of distance to the nearest chromosome end. Note that the scale of log_10_ p-values runs from 0 to -15 so that the height of the line corresponds to higher significance.
(TIF)

Figure S4 Methylation patterns for all genes. The average enrichment of H3K4me1 (blue), H3K4me2 (red) and H3K4me3 (grey) over H3, for the set of 5977 yeast genes that show at least a two-fold enrichment of H3K4me2 or H3K4me3 somewhere across the gene or flanking region [53].
(TIF)

Figure S5 H3K4 methylation patterns indicating antisense transcription. Patterns of H3K4 methylation [53] on the five model genes followed up in Figure 4B, expressed as log_2_ of each methylation mark over H3 (top panels). Mapping of coding regions by SGD indicated in red and non-coding ones by [39], indicated in blue and yellow for CUTs and SUTs, respectively (bottom panels).
(TIF)

Table S1 The 69 COMPASS-repressed genes and their distance from the nearest chromosome end.
(PDF)

Table S2 Evidence for the presence of ncRNAs in the COMPASS-repressed genes that have H3K4me2/3 levels more than 2-fold over H3. The evidence for non-coding transcription is based on [39], [40]. Three types of non-coding RNAs were reported: antisense transcripts spanning the body of the gene (antisense), transcripts in the promoter of the genes (promoter) and known non-coding transcripts (SGD). “No data available” indicates cases when the above studies didn't include the regions of specific genes in their results.
(PDF)

Table S3 The 69 COMPASS-repressed genes are enriched in specific Gene Ontology functional categories and transcription factor binding sites in their promoters as described by the Fraenkel lab - MacIssac (2006) BMC Bioinformatics. The number of co-occurences between functional categories and the repressed genes (Hits), the corresponding genes (Annotated Genes), the number of background hits, as well as the corresponding Bonferroni-corrected p-values (Cor. p-val) are reported.
(PDF)

Table S4 Strains and plasmids used in this study.
(PDF)

Table S5 Primers used in this study.
(PDF)
