# Supplementary figures and images for: Two Distinct Repressive Mechanisms for Histone 3 Lysine 4 Methylation through Promoting 3′-End Antisense Transcription
Source: PLoS Genet. 2012 Sep 20;8(9):e1002952. doi: 10.1371/journal.pgen.1002952 (PMC3447963; doi:10.1371/journal.pgen.1002952)

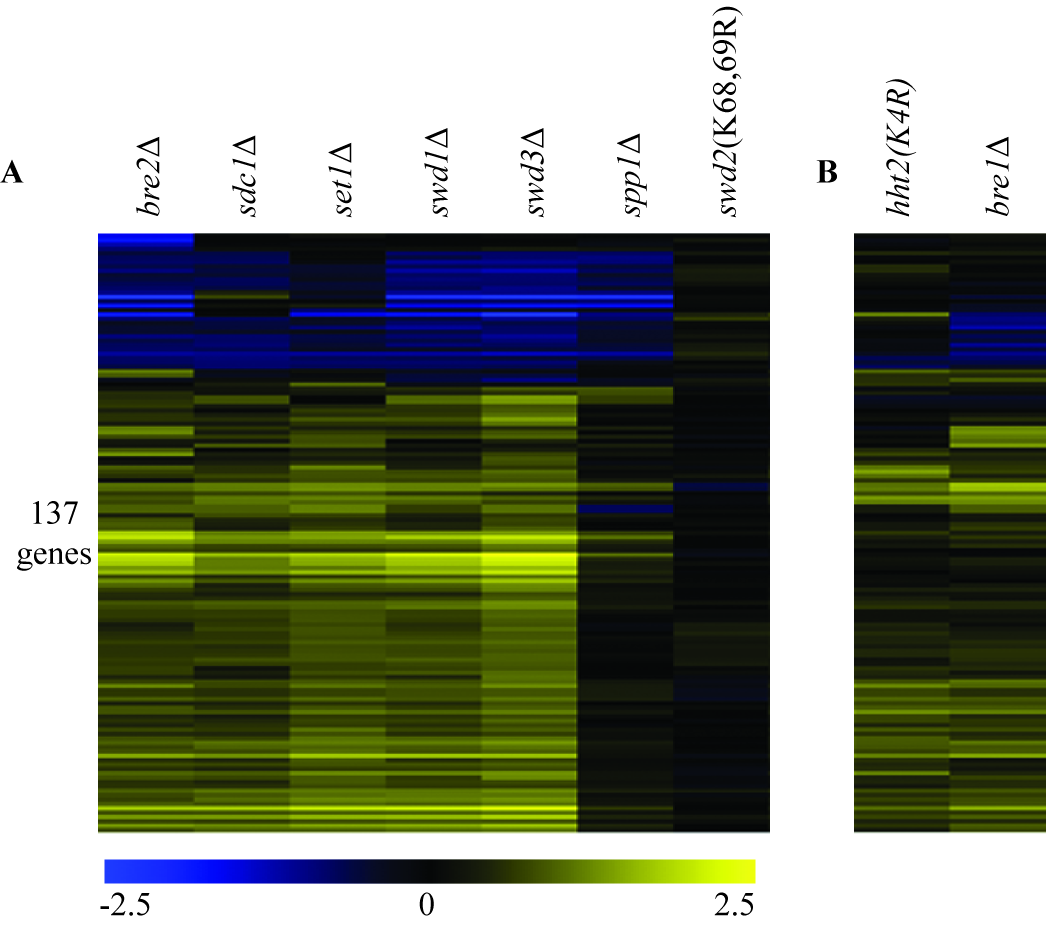

Supplement: Figure S1 — (A) Hierarchical clustering, as in Figure 1B, of all genes with significantly changed mRNA expression in any COMPASS mutant. Figure 1B depicts those genes that have significantly changed expression in at least two mutants. (B) Genes depicted in the same order as in A for the H3K4R point mutant and bre1Δ. (TIF) [file pgen.1002952.s001.tif]

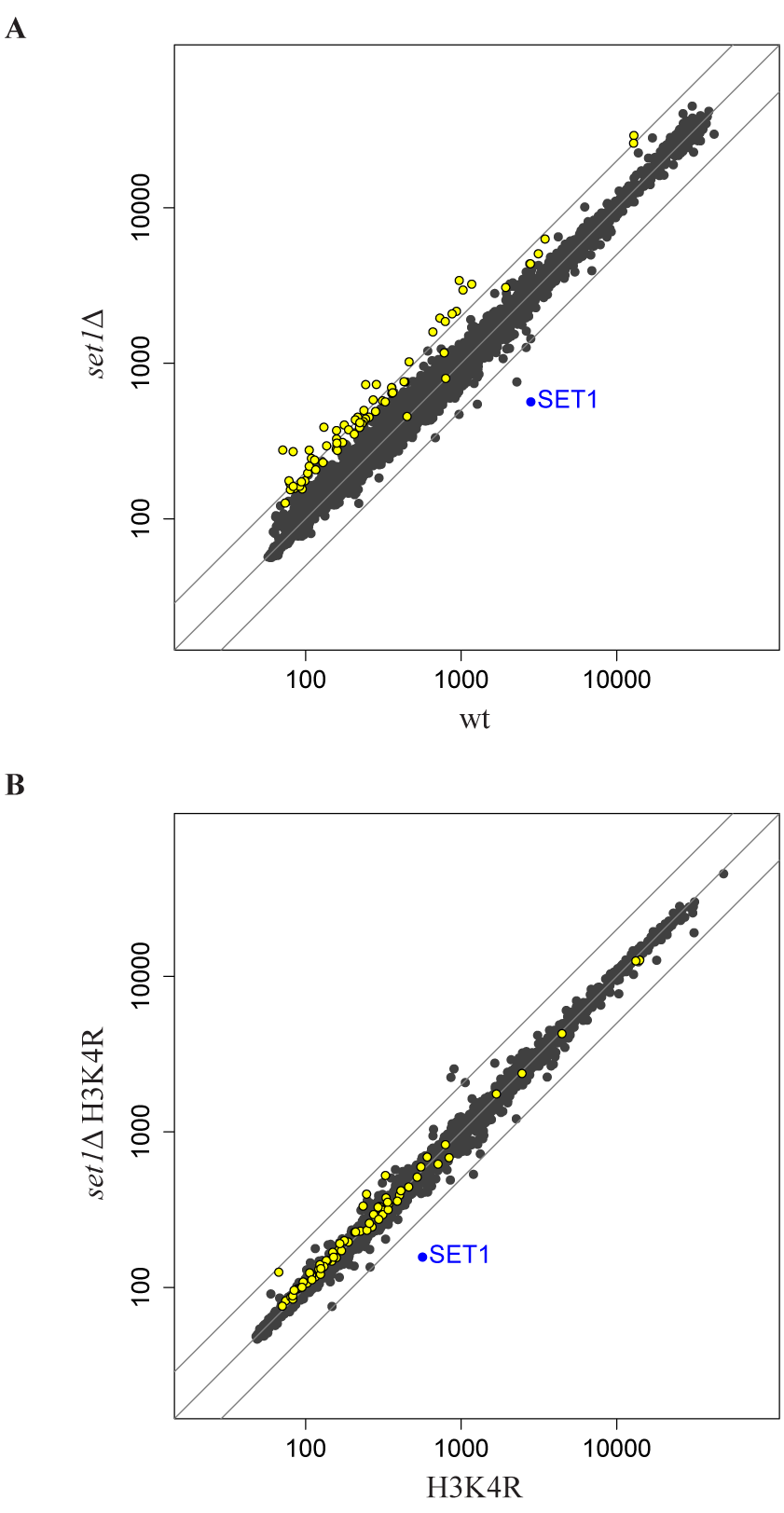

Supplement: Figure S2 — The repressive effect of Set1 on transcription is through H3K4 (A) Gene expression scatter plot of the average, normalized fluorescent intensity values of each gene in set1Δ compared to the wt strain. The 69 COMPASS-repressed genes are represented by yellow dots. (B) As in A, but now for the set1Δ H3K4R double mutant compared to the H3K4R point mutant. The deleted gene is represented by a blue dot. (TIF) [file pgen.1002952.s002.tif]

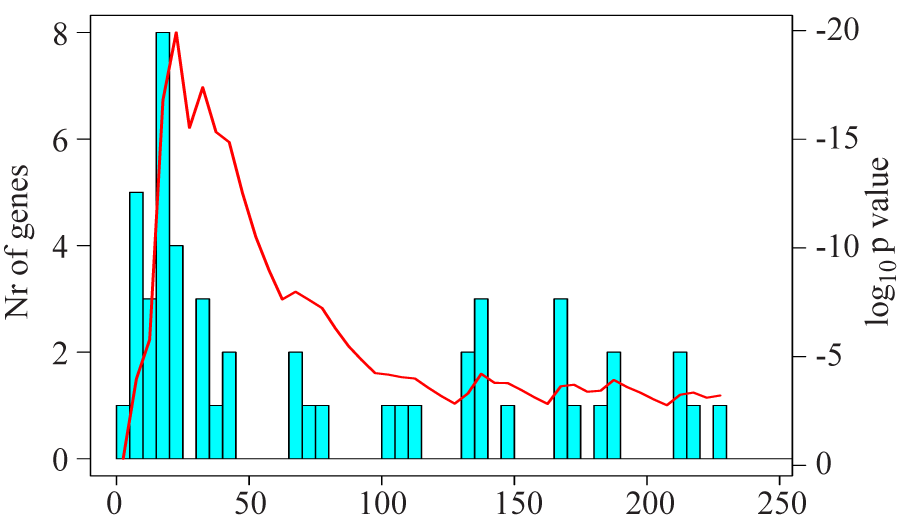

Supplement: Figure S3 — COMPASS-repressed genes are enriched near telomeres. The histogram shows the genomic location of the 69 genes significantly upregulated in at least two COMPASS deletion mutants. The bars represent the numbers of genes found in 5-kb intervals from nearest chromosome end. The line represents the log10 p-value as a function of distance to the nearest chromosome end. Note that the scale of log10 p-values runs from 0 to -15 so that the height of the line corresponds to higher significance. (TIF) [file pgen.1002952.s003.tif]

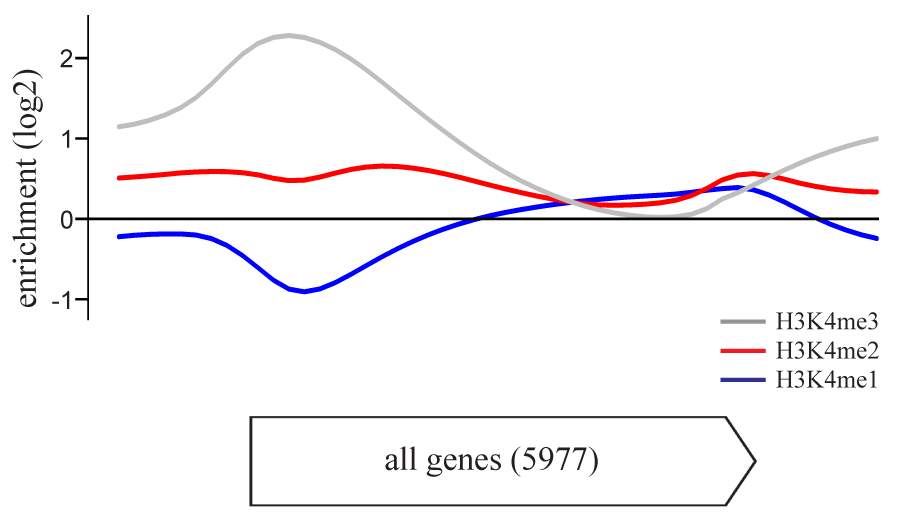

Supplement: Figure S4 — Methylation patterns for all genes. The average enrichment of H3K4me1 (blue), H3K4me2 (red) and H3K4me3 (grey) over H3, for the set of 5977 yeast genes that show at least a two-fold enrichment of H3K4me2 or H3K4me3 somewhere across the gene or flanking region [53]. (TIF) [file pgen.1002952.s004.tif]

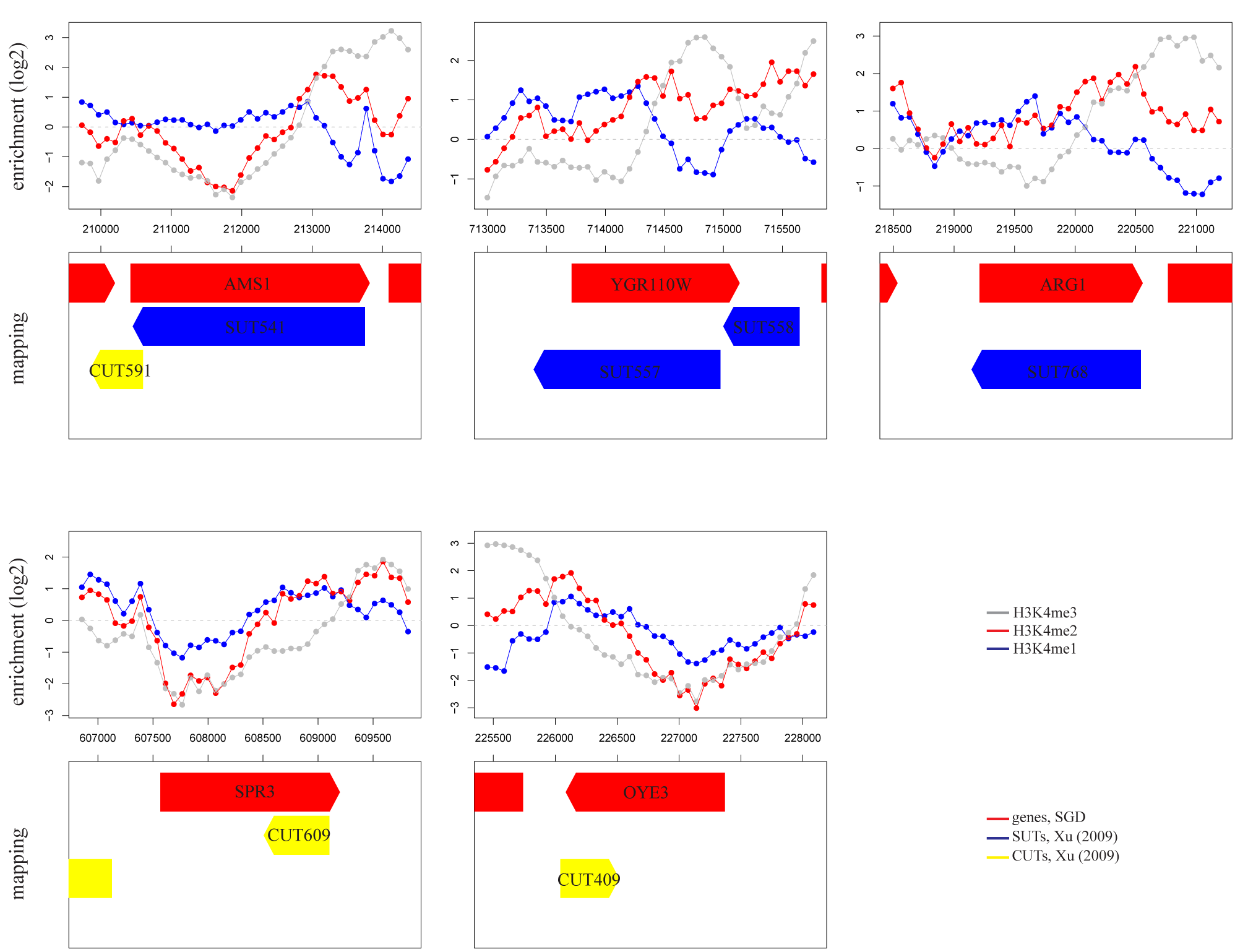

Supplement: Figure S5 — H3K4 methylation patterns indicating antisense transcription. Patterns of H3K4 methylation [53] on the five model genes followed up in Figure 4B, expressed as log2 of each methylation mark over H3 (top panels). Mapping of coding regions by SGD indicated in red and non-coding ones by [39], indicated in blue and yellow for CUTs and SUTs, respectively (bottom panels). (TIF) [file pgen.1002952.s005.tif]
