# Supplementary material for: Two Distinct Repressive Mechanisms for Histone 3 Lysine 4 Methylation through Promoting 3′-End Antisense Transcription
Source: PLoS Genet. 2012 Sep 20;8(9):e1002952. doi: 10.1371/journal.pgen.1002952 (PMC3447963; doi:10.1371/journal.pgen.1002952)
Supplement: Table S1 — The 69 COMPASS-repressed genes and their distance from the nearest chromosome end. (PDF) [file pgen.1002952.s006.pdf]

systematic name	gene symbol	Distance (bp)
YAR071W	PHO11	4056
YMR322C	SNO4	5709
YKL221W	MCH2	6819
YDL246C	SOR2	9220
YHR215W	PHO12	9843
YFL061W	DDI3	9884
YCR102C	YCR102C	11706
YPL280W	HSP32	12244
YFL058W	THI5	13440
YNL332W	THI12	15343
YBR296C	PHO89	15524
YJR156C	THI11	16666
YDL244W	THI13	16715
YJL221C	YJL221C	17652
YIL172C	YIL172C	17669
YPL276W	YPL276W	18167
YPL275W	YPL275W	18724
YDL241W	YDL241W	20821
YOL157C	YOL157C	23409
YIL169C	YIL169C	24613
YML123C	PHO84	24920
YOL155C	HPF1	30154
YIR027C	DAL1	32937
YNR064C	YNR064C	34761
YBL098W	BNA4	39833
YEL059C-A	SOM1	42512
YAR031W	PRM9	42930
YCL030C	HIS4	67134
YFR023W	PES4	69369
YCL026C-B	HBN1	73696
YGR260W	TNA1	77653
YJL170C	ASG7	101459
YDL196W	YDL196W	106907
YFL012W	YFL012W	110864
YGR234W	YHB1	130439
YGR233C	PHO81	134501
YHR015W	MIP6	135537
YBL043W	ECM13	137078
YLL005C	SPO75	139242
YKL162C-A	YKL162C-A	146003
YJR079W	YJR079W	165030
YJR078W	BNA2	166212
YMR244W	YMR244W	166647
YBR208C	DUR1,2	173727
YNL018C	YNL018C	183477
YHR137W	ARO9	186161
YHR136C	SPL2	187763
YGL156W	AMS1	212047
YHR053C	CUP1-2	212630
YHR055C	CUP1-2	214628
YOL058W	ARG1	219841
YPL171C	OYE3	226769
YDR403W	DIT1	256519
YER072W	VTC1	273871
YBR148W	YSW1	274394
YGR144W	THI4	310052
YBR040W	FIG1	317416
YBR045C	GIP1	329230
YBR047W	FMP23	332095
YLR307W	CDA1	332101
YBR115C	LYS2	341347
YJL037W	IRC18	368751
YJL038C	YJL038C	370306
YGR110W	YGR110W	376565
YDL039C	PRM7	382157
YDL038C	YDL038C	383203
YPL019C	VTC3	432300
YGR059W	SPR3	482610
YDR281C	PHM6	509758
