# Supplementary material for: Two Distinct Repressive Mechanisms for Histone 3 Lysine 4 Methylation through Promoting 3′-End Antisense Transcription
Source: PLoS Genet. 2012 Sep 20;8(9):e1002952. doi: 10.1371/journal.pgen.1002952 (PMC3447963; doi:10.1371/journal.pgen.1002952)
Supplement: Table S2 — Evidence for the presence of ncRNAs in the COMPASS-repressed genes that have H3K4me2/3 levels more than 2-fold over H3. The evidence for non-coding transcription is based on [39], [40]. Three types of non-coding RNAs were reported: antisense transcripts spanning the body of the gene (antisense), transcripts in the promoter of the genes (promoter) and known non-coding transcripts (SGD). “No data available” indicates cases when the above studies didn't include the regions of specific genes in their results. (PDF) [file pgen.1002952.s007.pdf]

systematic name	gene symbol	Xu(2009)	Neil(2009)	Type
YAR031W	PRM9	no data available	no data available	no data available
YAR071W	PHO11	likely	likely	antisense
YBL098W	BNA4	likely	yes	antisense
YBR040W	FIG1	not detected	not detected	not detected
YBR047W	FMP23	likely	yes	antisense
YBR115C	LYS2	yes	yes	promoter
YBR148W	YSW1	yes	yes	antisense
YBR208C	DUR1,2	not detected	not detected	not detected
YCL026C-B	HBN1	likely	likely	promoter
YCL030C	HIS4	yes	yes	promoter
YCR102C	YCR102C	yes	not detected	antisense
YDL039C	PRM7	yes	yes	antisense
YDL196W	YDL196W	likely	likely	antisense
YDL241W	YDL241W	yes	yes	antisense
YDR281C	PHM6	likely	yes	antisense
YDR403W	DIT1	yes	yes	antisense
YEL059C-A	SOM1	likely	likely	antisense
YER072W	VTC1	yes	yes	antisense and promoter
YFL012W	YFL012W	yes	yes	promoter and antisense
YFL061W	DDI3	no data available	no data available	no data available
YGL156W	AMS1	yes	yes	antisense and promoter
YGR059W	SPR3	yes	yes	antisense
YGR110W	YGR110W	yes	not detected	antisense and promoter
YGR144W	THI4	yes	yes	antisense and promoter
YGR233C	PHO81	yes	yes	promoter
YGR234W	YHB1	yes	yes	promoter
YGR260W	TNA1	yes	likely	promoter
YHR015W	MIP6	likely	likely	antisense
YHR053C	CUP1-1	no data available	not detected	ncRNA (SGD)
YHR055C	CUP1-2	no data available	not detected	ncRNA (SGD)
YHR136C	SPL2	not detected	likely	antisense
YHR137W	ARO9	yes	yes	antisense
YHR215W	PHO12	yes	yes	antisense and promoter
YIL169C	YIL169C	yes	yes	antisense
YIR027C	DAL1	yes	not detected	antisense
YJL170C	ASG7	yes	yes	antisense
YJR078W	BNA2	not detected	yes	antisense
YJR079W	YJR079W	not detected	yes	antisense
YKL221W	MCH2	yes	not detected	antisense
YLL005C	SPO75	not detected	likely	antisense
YML123C	PHO84	yes	yes	antisense and promoter
YMR244W	YMR244W	not detected	likely	antisense
YOL058W	ARG1	yes	yes	antisense
YOL155C	HPF1	yes	yes	antisense
YOL157C	YOL157C	yes	yes	antisense
YPL019C	VTC3	likely	yes	antisense and promoter
YPL171C	OYE3	yes	yes	antisense
