# Supplementary material for: Two Distinct Repressive Mechanisms for Histone 3 Lysine 4 Methylation through Promoting 3′-End Antisense Transcription
Source: PLoS Genet. 2012 Sep 20;8(9):e1002952. doi: 10.1371/journal.pgen.1002952 (PMC3447963; doi:10.1371/journal.pgen.1002952)
Supplement: Table S3 — The 69 COMPASS-repressed genes are enriched in specific Gene Ontology functional categories and transcription factor binding sites in their promoters as described by the Fraenkel lab - MacIssac (2006) BMC Bioinformatics. The number of co-occurences between functional categories and the repressed genes (Hits), the corresponding genes (Annotated Genes), the number of background hits, as well as the corresponding Bonferroni-corrected p-values (Cor. p-val) are reported. (PDF) [file pgen.1002952.s008.pdf]

GO-BP

Name	Cor. P-value	Hits	Backgr. Hits	Annotated Genes
thiamin biosynthetic process	6.22E-05	5	15	THI11, THI12, THI13, THI5, THI4
thiamin and derivative biosynthetic process	8.98E-05	5	16	THI11, THI12, THI13, THI5, THI4
thiamin metabolic process	1.26E-04	5	17	THI11, THI12, THI13, THI5, THI4
thiamin and derivative metabolic process	1.73E-04	5	18	THI11, THI12, THI13, THI5, THI4
vitamin biosynthetic process	4.22E-04	7	58	THI11, BNA2, BNA4, THI12, THI13, THI5, THI4
water-soluble vitamin biosynthetic process	4.22E-04	7	58	THI11, BNA2, BNA4, THI12, THI13, THI5, THI4
vitamin metabolic process	2.05E-03	8	102	SNO4, THI11, BNA2, BNA4, THI12, THI13, THI5, THI4
response to copper ion	2.17E-03	3	5	CUP1-2, CUP1-1, YCR102C
cellular aromatic compound metabolic process	4.17E-03	9	147	ARO9, ARG1, THI12, HIS4, THI11, THI13, THI5, DAL1, THI4
aromatic compound biosynthetic process	6.57E-03	5	36	THI11, THI12, THI13, THI5, THI4
water-soluble vitamin metabolic process	1.39E-02	7	98	THI11, BNA2, BNA4, THI12, THI13, THI5, THI4
polyphosphate metabolic process	1.77E-02	3	9	VTC1, VTC3, PHO84
ascospore wall assembly	2.20E-02	5	46	CDA1, SPO75, DIT1, GIP1, SPR3
spore wall assembly	2.20E-02	5	46	CDA1, SPO75, DIT1, GIP1, SPR3
spore wall biogenesis	2.20E-02	5	46	CDA1, SPO75, DIT1, GIP1, SPR3
ascospore wall biogenesis	2.20E-02	5	46	CDA1, SPO75, DIT1, GIP1, SPR3
cell wall assembly	2.44E-02	5	47	CDA1, SPO75, DIT1, GIP1, SPR3
maltose metabolic process	3.43E-02	3	11	YOL157C, FSP2, YIL172C
heterocycle biosynthetic process	3.60E-02	6	80	THI11, THI12, THI13, THI5, THI4, HIS4
cellular nitrogen compound metabolic process	4.25E-02	13	396	LYS2, ARO9, ARG1, THI12, HIS4, DUR1,2, THI11, BNA2, BNA4, THI13, THI5, THI4, DAL1
response to toxin	4.37E-02	4	29	YHB1, THI13, THI5, YNR064C

GO-MF

Name	Cor. P-value	Hits	Backgr. Hits	Annotated Genes
alpha-glucosidase activity	4.67E-03	3	8	YOL157C, FSP2, YIL172C
glucosidase activity	6.71E-03	4	23	YOL157C, FSP2, YIL172C, HPF1
hydrolase activity, hydrolyzing O-glycosyl compounds	7.77E-03	5	45	YOL157C, AMS1, FSP2, YIL172C, HPF1
hydrolase activity, acting on glycosyl bonds	1.71E-02	5	53	YOL157C, AMS1, FSP2, YIL172C, HPF1
formate dehydrogenase activity	2.50E-02	2	3	YPL276W, FDH2
oxidoreductase activity	3.89E-02	11	322	LYS2, YHB1, YPL276W, HIS4, BNA2, BNA4, OYE3, SOR2, YCR102C, HBN1, FDH2

GO-CC

Name	Cor. P-value	Hits	Backgr. Hits	Annotated Genes
vacuolar transporter chaperone complex	3.31E-02	2	4	VTC1, VTC3

TFBS (Fraenkel)

Name	Cor. P-value	Hits	Backgr. Hits	Annotated Genes
SUM1	6.29E-05	8	66	TNA1, PES4, BNA2, YSW1, SPO75, DAL1, SPR3, PHM6
PHO4	1.88E-04	6	34	VTC3, ARO9, PHO84, PHO89, SPL2, PHM6
ARO80	1.43E-02	2	2	ARO9, SPL2
