# Supplementary material for: Two Distinct Repressive Mechanisms for Histone 3 Lysine 4 Methylation through Promoting 3′-End Antisense Transcription
Source: PLoS Genet. 2012 Sep 20;8(9):e1002952. doi: 10.1371/journal.pgen.1002952 (PMC3447963; doi:10.1371/journal.pgen.1002952)
Supplement: Table S4 — Strains and plasmids used in this study. (PDF) [file pgen.1002952.s009.pdf]

Name	Genotype	Source
bre1Δ	*MAT*a *his3Δ1 leu2Δ0 met15Δ0 ura3Δ0 bre1Δ::KANR*	Euroscarf
bre2Δ	*MAT*a *his3Δ1 leu2Δ0 met15Δ0 ura3Δ0 bre2Δ::KANR*	Euroscarf
hht1Δhhf1Δ	*MATa his3Δ200 leu2Δ0 lys2Δ0 trp1Δ63 ura3Δ0 met15Δ0 hht1-hhf1::NatMX4 hhf2::HHTS/HHFS-URA3can1::MFA1pr-HIS3*	(Dai et al. 2008)
rrp6Δ	*MAT*a *his3Δ1 leu2Δ0 met15Δ0 ura3Δ0 rrp6Δ::HPH*	this study
sdc1Δ	*MAT*a *his3Δ1 leu2Δ0 met15Δ0 ura3Δ0 sdc1Δ::KANR*	Euroscarf
set1Δ	*MAT*a *his3Δ1 leu2Δ0 met15Δ0 ura3Δ0 set1Δ::KANR*	this study
set1Δrrp6Δ	*MATa his3Δ1 leu2Δ0 met15Δ0 ura3Δ0 set1Δ::KANR rrp6Δ::HPH*	this study
spp1Δ	*MAT*a *his3Δ1 leu2Δ0 met15Δ0 ura3Δ0 spp1Δ::KANR*	Euroscarf
swd1Δ	*MAT*a *his3Δ1 leu2Δ0 met15Δ0 ura3Δ0 swd1Δ::KANR*	Euroscarf
swd2Δ pSWD2	*MAT*a *his3Δ1 leu2Δ0 met15Δ0 ura3Δ0 swd2Δ::KANR p415-LEU-SWD2-HA*	(Vitaliano-Prunier et al. 2008)
swd2Δ pSWD2K68,69R	*MAT*a *his3Δ1 leu2Δ0 met15Δ0 ura3Δ0 swd2Δ::KANR p415-LEU- SWD2 K68,69R-HA*	(Vitaliano-Prunier et al. 2008)
swd3Δ	*MAT*a *his3Δ1 leu2Δ0 met15Δ0 ura3Δ0 swd3Δ::KANR*	Euroscarf
wt	*MAT*a *his3Δ1 leu2Δ0 met15Δ0 ura3Δ0*	Euroscarf
wt reference	*MATα his3Δ1 leu2Δ0 lys2Δ0 ura3Δ0*	Euroscarf
yAM212	*MATa ura3-52, his3Δ20, leu2Δ, trp1Δ63 lys−128Δ, (hht1−hhf1)::LEU2; (hht2−hhf2)::HIS3 pRS314-HHT2-HHF2*	(Berretta et al. 2008)
yAM213	*MATa ura3-52, his3Δ20, leu2Δ, trp1Δ63 lys−128Δ, (hht1−hhf1)::LEU2; (hht2−hhf2)::HIS3 pRS314-hht2K4R-HHF2*	A. Morrillon lab
yAM213 set1Δ	*MATa ura3-52, his3Δ20, leu2Δ, trp1Δ63 lys−128Δ, (hht1−hhf1)::LEU2; (hht2−hhf2)::HIS3 pRS314-hht2K4R-*	this study

	HHF2 set1Δ::KANR	
YGR110W-ingdel SET1		this study
YGR110W-ingdel set1Δ		this study
