# Supplementary material for: Two Distinct Repressive Mechanisms for Histone 3 Lysine 4 Methylation through Promoting 3′-End Antisense Transcription
Source: PLoS Genet. 2012 Sep 20;8(9):e1002952. doi: 10.1371/journal.pgen.1002952 (PMC3447963; doi:10.1371/journal.pgen.1002952)
Supplement: Table S5 — Primers used in this study. (PDF) [file pgen.1002952.s010.pdf]

Primer Name	Primer sequence 5'->3'
**RT Primers**	
5’RT AMS1 AS	GGGTAGAATACCTGGACACG
3’RT AMS1 S	TTTGATTCGTCTCGCGTTGC
5’RT YGR110W AS	GCTTGTTTCCTCGCCCTTTA
3’RT YGR110W S	GATGCCCTGAATGAGGAACA
5’RT ARG1 AS	GGAAAAGGCCTTGAAGATCG
3’RT ARG1 S	GCTATTTTGGGATGGTTGGA
5’RT SPR3 AS	CGATGAAATCGAGGAAAACAA
3’RT SPR3 S	GTGGAATGGCTGTTGGAACT
5’RT OYE3 AS	AGAGGGCAAAGGCTGGTAAG
3’RT OYE3 S	TGGGTAGTCGGTATAACCTTCC
**qPCR Primers**	
5’qAMS1	GGGTTTTCCACACACGGTAA
3’qAMS1	TTCGTGAGCGAGCTTAACAG
5’qYGR110W	TTCACAGCGTTCAAGATTGG
3’qYGR110W	GGGGAACAAAGGATCAGCTT
5’qARG1	AGATTTGCTGGCAGAAAGGA
3’qARG1	CTTTGGTGGGGTGGTATCTG
5’qSPR3	AAAAATTCAACGAGCTGTCCA
3’qSPR3	ATTCCAAGTCCTGACATTCCA
5’qOYE3	ATGGAAGGGTCCAATCATCA
3’qOYE3	ATGGCAGGCCCTCTTCTAAA
**Constructs Primers**	
YGR110W_HIS5_F	ACTATAAGTTTTTCAATAATATTCTTACGAAAGAAATGCAAAAAATAT AAGCATAGGCCACTAGTGGATCTG
YGR110W_HIS5_R	TCGCTATAACATTCGAATATTAGGTCATCGTCATTTGATGAGGCTGT CATCAGCTGAAGCTTCGTACGC
